# Structure Characterization and Treatment Effect of *Zingiber officinale* Polysaccharide on Dextran Sulfate Sodium-Induced Ulcerative Colitis

**DOI:** 10.3390/foods14050753

**Published:** 2025-02-23

**Authors:** Yongshuai Jing, Ziying Wang, Wenjing Cheng, Hanju Fan, Kaiyan Zheng, Yuguang Zheng, Lanfang Wu

**Affiliations:** 1 College of Chemical and Pharmaceutical Engineering, Hebei University of Science and Technology, 26 Yuxiang Street, Shijiazhuang 050018, China; cjys1985@126.com (Y.J.); ziyingwang@hebust.edu.cn (Z.W.); wenjingcheng@hebust.edu.cn (W.C.); hanjufan@hebust.edu.cn (H.F.); 2 College of Pharmacy, Hebei University of Chinese Medicine, 3 Xingyuan Road, Shijiazhuang 050200, China; zhengkaiyan168@126.com

**Keywords:** *Zingiber officinale*, ZOP-1, UC, gut microbiota, immunohistochemistry, Western blot

## Abstract

Background: Ulcerative colitis (UC) is on the rise all over the world. *Zingiber officinale* polysaccharide (ZOP-1) has good anti-inflammatory and antioxidant effects, but the therapeutic effect and mechanism of ZOP-1 on UC are still unclear. Methods: ZOP-1 obtained by water extraction and alcohol precipitation was analyzed by methylation and NMR. At the same time, the mechanism of ZOP-1 in the treatment of UC was clarified by hematoxylin-eosin (HE) staining, metagenomics, immunohistochemistry, and protein blot (Wb). Results: ZOP-1 was the structure of the by →4,6)-β-Glcp-1→ and →3,6)-α-Galp-(1→ constitute the main chain, there were two branched chain by →4)-β-Glcp(1→, and α-Araf(1→ as the end group. ZOP-1 significantly improved the shortening and thickening of the colon, changed the index of immune organs, inhibited the production of inflammatory factors in mice with ulcerative colitis, changed the intestinal flora of mice, increased the content of short-chain fatty acids (SCFAs) in the intestine, and controlled the TLR4/NF-κB/MAPK signaling pathway, thus preventing and treating DSS-induced ulcerative colitis in mice. Conclusions: ZOP-1 alleviated UC by controlling the expression of cytokines, thereby reducing intestinal inflammation and oxidative stress, enhancing intestinal integrity, modulating intestinal flora, and regulating the levels of SCFAs.

## 1. Introduction

Ulcerative colitis (UC) is a kind of pathological change involving colorectal mucosa of chronic, nonspecific inflammatory bowel disease, pathogenic factors often involve genetic susceptibility, intestinal environment, and other external medium mutual interference [1]. The complex pathophysiology of UC may be related to immune regulation, intestinal flora, heredity, environment, and psychological variables, and it is easy to recur, which seriously affects people’s quality of life, and there is no thorough treatment [2,3]. A great deal of research has confirmed that the imbalance of intestinal flora is highly correlated with UC [4]. Metabolites derived from intestinal flora, especially short-chain fatty acids (SCFAs), can significantly affect immune balance and maintenance of mucosal integrity [5]. At the same time, a large range of drugs can be used to treat UC, such as corticosteroids, 5-aminosalicylic acid, biological agents, and immunosuppressant drugs, but due to the high price of drugs, serious toxic and side effects may occur during the use of drugs [6]. Therefore, to solve the above problems, it is imperative to develop a new type of safe drug.

Natural products show excellent activity in the treatment of ulcerative colitis and have the characteristics of less toxic and side effects. Among them, *Zingiber officinale* Roscoe is a common medicinal and edible resource, and its roots and leaves can be used as medicine. It is rich in resources in China and has a long planting history. Ginger contains many active components, such as volatile oil, curcumin, flavonoids, and polysaccharides [7]. Polysaccharide is one of the effective components of *Zingiber officinale* Roscoe, which has anti-inflammatory, immunomodulatory, antioxidant, anti-tumor, regulating intestinal flora and other biological activities [8]. Studies have shown that polysaccharides from different sources have effects on UC, such as *Houttuynia cordata* polysaccharide had a good anti-ulcerative colitis effect [9], and *pine pollen* polysaccharide inhibited the secretion of proinflammatory cytokines, increased the secretion of related anti-inflammatory cytokines, and adjusted the dynamic balance of intestinal flora, thus achieving the improvement effect on DSS-induced UC mice [10]. Therefore, polysaccharide, as an important active substance, has been widely concerned in the treatment of UC. However, the mechanism of ZOP-1 on UC is still unclear.

In this study, the crude polysaccharide ZOP was successfully extracted from *Zingiber officinale* Roscoe, and the purified polysaccharide ZOP-1 was obtained by separation and purification. In this paper, the structure of ZOP-1 was characterized. On this basis, the influence of ZOP-1 on UC was investigated by endoscopic observation, colon length and thickness, immune organ index, inflammatory factors, and other indicators. At the same time, the effect of ZOP-1 on intestinal flora and the increase in short-chain fatty acids in mice were discussed in order to explore the role of intestinal flora in anti-UC. The purpose of this study is to explore the effect of ZOP-1 on intestinal flora and biochemical indexes related to UC, and to understand its anti-UC mechanism, so as to provide the basis for the subsequent development of functional food or healthcare products against ulcerative colitis.

## 2. Material and Methods

### 2.1. Materials and Chemicals

The supplier of *Z. officinale* (No. 2019031401) was Anguo Yaoyuan Trading Co., Ltd. (Baoding, China) Professor Lanfang Wu (Hebei University of Chinese Medicine) recognized it as the rhizome of *Zingiber officinale* Roscoe. Shanghai Aladdin Biochemical Technology Co., Ltd. (Shanghai, China) was the supplier of dextran sulfate sodium (DSS, Mw: 40 kDa. DSS was used to induce ulcerative colitis in mice.), dimethyl sulfoxide (DMSO), trifluoroacetic acid (TFA), acetonitrile (chromatographic grade), methanol (chromatographic grade) and 1-Phenyl-3-methyl-5-pyrazolone (PMP). DEAE cellulose-52 was purchased from Beijing Biotopped Science& Technology Co., Ltd. (Beijing, China). Malondialdehyde (MDA), Superoxide Dismutase, (SOD), myeloperoxidase (MPO), interleukin-1β (IL-1β), tumor necrosis factor-α (TNF-α), and interleukin-6 (IL-6) were purchased from Nanjing Jiancheng Bioengineering Institute (Nanjing, China). TLR4, MyD88, NF-κB, P-NF-κB, P38, and P-P38 antibodies were purchased from Shanghai Beyotime Biotechnology Co., Ltd. (Shanghai, China).

### 2.2. Extraction and Purification of ZOP-1

**Figure 1:**
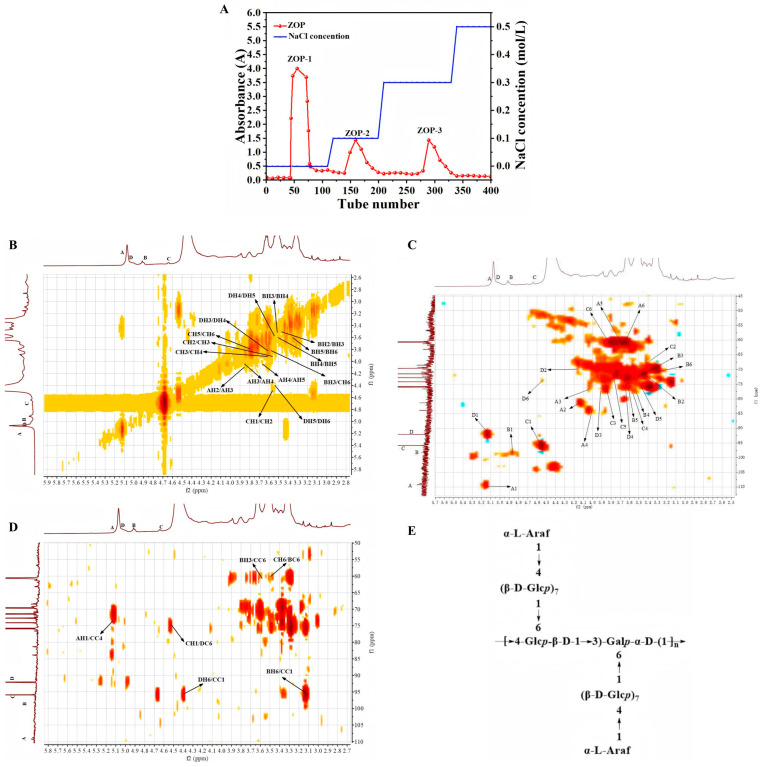
(**A**) Elution curve, (**B**) H-H COSY, (**C**) HSQC, (**D**) HMBC, and (**E**) predicted structure of ZOP-1.

Following the earlier research, the ZOP was extracted [9]. *Zingiber officinale* Roscoe (100 g) was taken, 95% ethanol was added according to the ratio of material to liquid of 1:3, and the mixture was heated and refluxed twice at 80 °C for 2 h each time for degreasing. Then, defatted *Zingiber officinale* Roscoe (50 g) was taken, distilled water was added according to the ratio of material to liquid of 1:30 and extracted twice at 90 °C for 2 h each time to obtain ZOP. Subsequently, ZOP (500 mg) was taken, and an appropriate amount of distilled water was added to completely dissolve it under ultrasonic waves. The solution was centrifuged for 10 min at 3000 rpm, and the supernatant was collected, and filtered with a filter membrane. Finally, it was passed through a DEAE-52 cellulose column by elution with distilled water, 0.1 M, 0.3 M, and 0.5 M NaCl solution in turn. The washed polysaccharide was named ZOP-1. The elution diagram of ZOP separation and purification is shown in Figure 1A.

### 2.3. Methylation Analysis of ZOP-1

ZOP-1 (2 mg) was dissolved in 1 mL of dimethyl sulfoxide, and anhydrous sodium hydroxide was added. Dissolve the mixture completely with an ultrasonic wave. Then, 1.0 mL of 98% CH_3_I was added and reacted in a magnetic stirring water bath at 30 °C for 60 min. The methylated polysaccharides were hydrolyzed by TFA (2 M) for 90 min and evaporated by rotary evaporator. 2 mL of double distilled water was added to the residue, 60 mg of sodium borohydride was reduced for 8 h, glacial acetic acid was added for neutralization, blew dry with nitrogen, then 1 mL of acetic anhydride was added for acetylation at 100 °C for 1 h and then cooled. Then, excess acetic anhydride was removed with toluene. Then, the acetylation product was extracted repeatedly with CH_2_Cl_2_ for 4 times, the organic layer was taken, the excess water was removed, the volume was fixed, and the sample was determined by gas chromatography-mass spectrometry (GC-MS). GC-MS conditions: RXI-5 SIL MS column 30 m × 0.25 mm × 0.25 μm; The temperature programmed conditions were as follows: the initial temperature was 120 °C, and the temperature was raised from 3 °C/min to 250 °C/min; The detector temperature was 250 °C/min, the carrier gas was He, and the flow rate was 1 mL/min.

### 2.4. Nuclear Magnetic Resonance (NMR) Spectrum Analysis of ZOP-1

30 mg of the sample was weighed and dissolved in D_2_O, and then the sample was analyzed at 25 °C with a 500 MHz spectrometer (Brook, BW Germany) to generate 1D and 2D NMR spectra [11].

### 2.5. Animal Experiments

**Figure 2:**
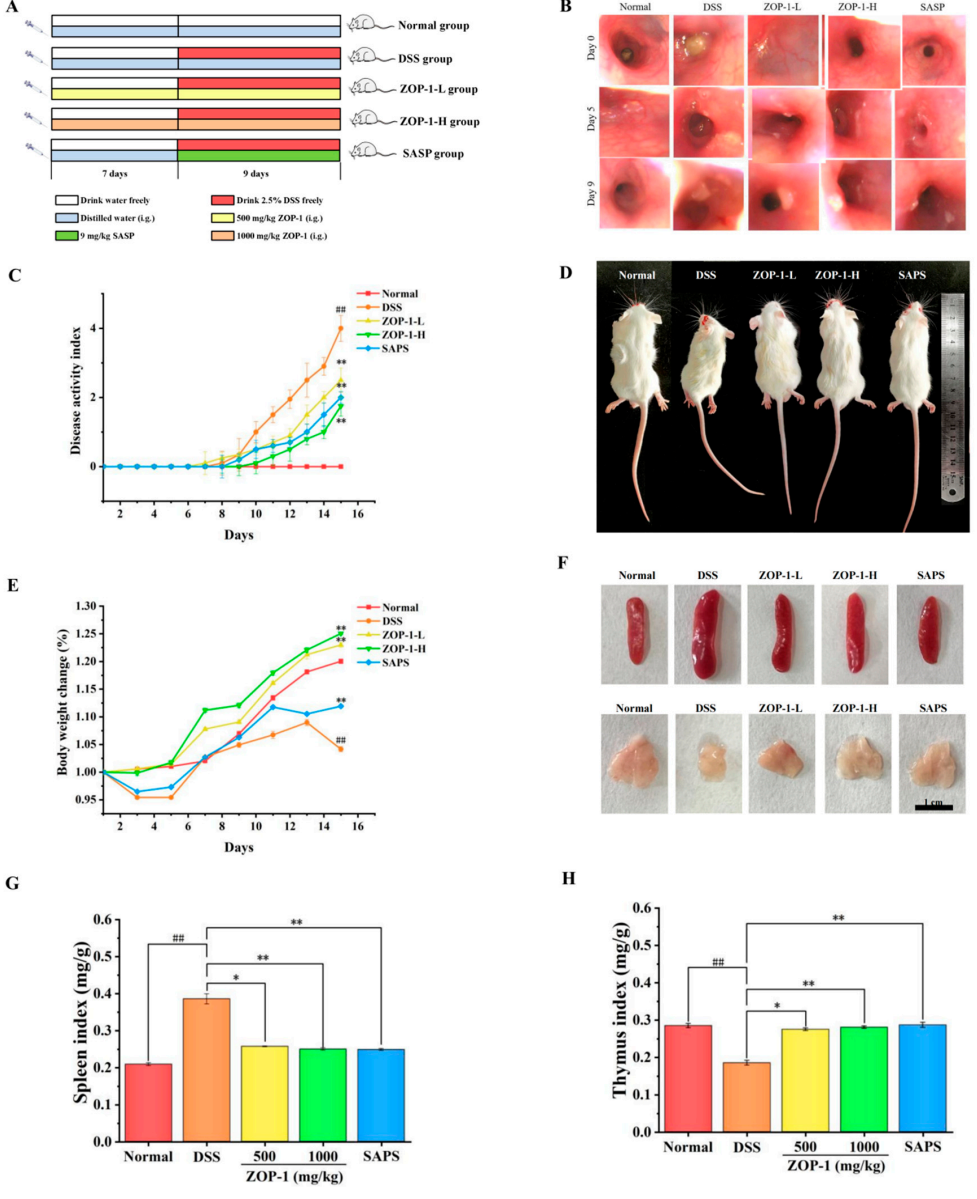
(**A**) Diagram schematic for the experiment on animals; (**B**) Picture of mouse colon endoscope; (**C**) DAI; (**D**) representative body images of mice in each group; (**E**) weight; (**F**) photos of spleen and thymus; (**G**) spleen index; (**H**) thymus index; In comparison to the Normal group, ## *p* < 0.01; in comparison to the DSS group, * *p* < 0.05, ** *p* < 0.01 (n = 6).

Eight-week-old healthy male mice could freely obtain common food and water under controlled conditions (60 ± 5% humidity and 23 ± 2 °C). This study involved the following experimental groups: control group (Normal), model control group (DSS), low-dose polysaccharide (ZOP-1-L), high-dose polysaccharide (ZOP-1-H), and positive control group (Sulfasalazine enteric-coated tablets, SAPS), Six mice in each group. After 7 days of adaptive feeding, the model was established. Mice in the Normal group drank distilled water freely, while other groups drank 2.5% DSS. The DSS group was given distilled water, while the positive control group was given 9 mg/kg SASP, 500 mg/kg ZOP-1 in the low-dose group, and 1000 mg/kg ZOP-1 in the high-dose group. The modeling time was 9 days. The specific modeling method is shown in Figure 2A. Beijing Huafukang Biotechnology Co., Ltd. (Beijing, China) provided the Kunming (KM) mouce (20.0 ± 2.0 g) (Certificate number: [SCXK (Beijing) 2019-0008]). The National Institutes of Health’s Guidelines for the Care and Use of Laboratory animals were closely followed in the treatment of the animals, and the Hebei University of Science and Technology’s Animal Care and Use Ethics Review Committee approved all experimental procedures (SCXK (Ji) 2020-002, NO.110322220100608582).

### 2.6. DAI Score of UC Model

By referring to the literature [12], the body weight, fecal viscosity, and hematozoa of the mouse after modeling were scored, and DAI scores were taken every 2 days to evaluate the severity of colitis. Each index of the score was scored 0–4 points. DAI score was calculated by the following conditions: diarrhea (0, normal; 2, loose stools; 4, watery diarrhea; Blood in the stool (0, no bleeding; 2, slight bleeding; 4, massive hemorrhage) and weight loss (0, ≤1%; 1–5%; 2, 5–10%; 3, 10–15%; 4, >15%).

### 2.7. Spleen and Thymus Index

In order to determine the organ index, the thymus, and spleen were taken out after euthanasia with anesthesia and their weights were recorded.

### 2.8. Colon Length and Histopathological Observation in Mouce

The mice were euthanized under anesthesia, and colon tissue was dissected out and measured for length and thickness. The histological changes in the colon were analyzed by hematoxylin and eosin (HE) staining.

### 2.9. Biochemical Index Determination in Serum and Tissues

The contents of SOD, MDA, MPO, IL-1β, TNF-α, and IL-6 in serum and tissues were determined according to the instructions of the ELISA kit [13].

### 2.10. Metagenomic Analysis

The effects of ZOP-1 on intestinal microbiota in mice were detected by metagenome. The feces of mouce were collected and stored in a −80 °C refrigerator. Sample sequencing and bioinformatics were conducted by Guangzhou Gene Denovo Biotechnology Co., Ltd. (Guangzhou, China). The effects of ZOP-1 on bacterial communities were investigated by dilution curves, α and β diversity, and community function prediction.

### 2.11. Calculating the SCFAs Content of Cecal Samples

Following the collection of the feces, they were freeze-dried and combined with ultra-pure water. The content of various SCFAs was ascertained by taking the supernatant following centrifugation, ultrasonography, and PH adjustments [14].

### 2.12. Protective Effect of ZOP-1 on Intestinal Epithelial Cells in Mice with UC

Occludin and zonula occludens protein-1 (ZO-1) expression in the colon was evaluated by immunohistochemical analysis [15]. They were incubated with non-specific staining-blocking reagents, and then the sections were incubated with the corresponding secondary antibody. Photos were taken with the DM2500 optical microscope.

### 2.13. Determination of Related Pathway Proteins by Western Blot

The protein expressions of TLR4, MyD88, and NF-κB in colon tissues were found using Western blot [16].

### 2.14. Statistical Analysis

SPSS 25.0 was used for statistical analysis; one-way analysis of variance (ANOVA) was used and LSD was used for post-analysis. Drawing with Origin 2021 software. The experimental values were displayed as mean ± SD. *p* < 0.05 was considered statistically significant.

## 3. Results

### 3.1. Methylation Results of ZOP-1

Previous studies have shown that the molecular weight of ZOP-1 was 83.72 kDa, and the monosaccharide composition was glucose, galactose, and arabinose = 85.09:5.00:9.91 [17].

Methylation and GC-MS were used to detect the sugar residue composition of ZOP-1, and the results are shown in Table 1. ZOP-1, mainly consists of four types of sugar chains, namely, 1-linked Araf, 1,4-linked Glcp, 1,4,6-linked Glcp, and 1,3,6-linked Galp residues. The results showed that ZOP-1 had a main chain primarily made up of 1,4,6-linked α-Glcp and 1,3,6-linked α-Galp, branching from C-6. The results of the type and proportion of glycosidic bonds were in line with those of monosaccharide analysis.

### 3.2. NMR Analysis of ZOP-1

Previous research results show that ZOP-1 has four different types of heterogeneous carbon signals, which are 5.15/108.99(A), 4.85/97.73(B), 4.52/95.78(C) and 5.12/91.86(D)ppm respectively [17]. According to previous literature [18,19,20], NMR data corresponding to the four glycosidic bonds are summarized in Table 2. Based on the findings of methylation analysis, there was a Galp residue in the repeat unit of ZOP-1. A chemical shift of 91.86/5.12 ppm was assigned to C1/H1 of →3,6)-β-Galp-1→ (residue D).

Combined with COSY (Figure 1B) and HSQC (Figure 1C) spectra, the proton chemical shifts in C/H were 91.86/5.12, 69.71/4.24, 77.75/3.89, 74.11/3.61, 74.29/3.54 and 75.22/4.40 ppm, respectively. Consequently, →3,6)-α-Galp(1→ was hypothesized to be residue D. Residue A had an ectopic proton signal of δ5.15 ppm and an ectopic carbon signal of δ108.99 ppm. According to the results of methylation analysis, two Glcp residues were found in the repeat unit of ZOP-1. Chemical shifts of 97.73/4.85 and 95.78/4.52 ppm were assigned to C1/H1 of →4)-β-Glcp(1→ (residue C) and →4,6)-β-Glcp(1→ (residue B), respectively. Meanwhile, the rest were signals of other residues. HSQC showed that other corresponding CH signals were 4.10/82.15, 3.88/79.45, 4.03/82.77, and 3.67/62.20 ppm, respectively. The presence of α-Araf-(1→) was suggested by the C1/H1 signal at 108.99/5.15 ppm.

Cross peaks between some residues were found in COSY spectra (Figure 1B) and HMBC spectra (Figure 1D), such as AH1 to CC4, CH1 to DC6 DH6 to CC1, etc. The cross-peak of 5.15/76.92 ppm was attributed to AH1/CC4 between residues, indicating that residues A and C were interconnected in the form of A-(1→4)-C. Furthermore, the signal at 4.52 ppm indicated the position of H1 in residue C, while the signal at 75.22 ppm indicated the position of C6 in residue D. It could be deduced that D-(6→1)-C was the connecting method between residues C and D.

The results concluded that ZOP-1 was the structure of the by →4,6)-β-Glcp-1→ and →3,6)-α-Galp-(1→ constitute the main chain, there were two branched chain by →4)-β-Glcp(1→ and α-Araf(1→ as the end group (Figure 1E). Previous studies have shown that the structure of ginger polysaccharide (ZOP-1) eluted with 0.1 mol/L NaCl was composed of → 3,4)-α-GLcp → and →4,6)-α-GLcp-(1 → as the main chain, →4)-α-Glcp (1→ as the side chain [21].

### 3.3. Endoscopic Changes and DAI Score in UC Mice

Each group contained 6 KM mouce. The animal experiment scheme is shown in Figure 2A. Compared with the other groups, mice in the DSS group showed slow weight gain, semi-dilute stool, fecal occult blood, anal bleeding, and other symptoms after 6 days of oral administration of 2.5% DSS solution. Intestinal damage in mice with UC was monitored by the endoscope. Figure 2B showed the changes in the colonic lining of mice in the Normal group, DSS group, ZOP-1 group, ZOP-1-H group, and SASP group on days 7–16. Observation on the 7th day showed that the colonic epithelium mucosa of the mice was normal, and no damage was found. On the 12th day, the DSS group, ZOP-1-L group, ZOP-1-H group, and SASP group all had different degrees of mucosal exfoliation. After comparison, it was found that the DSS group had the most serious mucosal exfoliation. In the next four days, the colons of mice in DSS, ZOP-1-L, ZOP-1-H, and SASP groups all showed thickened intestinal walls, unclear blood vessels, and mucous membrane shedding, among which the colonic wall of mice in DSS group showed large hemorrhagic spots, while that in ZOP-1-L group showed small hemorrhagic spots. No hemorrhagic sites were found in the colon wall of mice in the ZOP-1-H and SASP groups. It may be that DSS carries a “high negative charge contributed by sulfate groups” and may have damaging effects on colonic epithelium due to its “toxicity”, leading to the “erosion” of the mucosal epithelial barrier [22].

To evaluate the degree of colon injury, the Mayo endoscopic score [23] was used for comparison (Table S1). The Mayo scores of mice with UC in the Normal group, DSS group, ZOP-1-L group, ZOP-1-H group, and SASP group were 0, 3, 2, 1, and 1, respectively. Compared with the DSS group, ZOP-1-L, ZOP-1-H, and SASP groups alleviated the colon injury of DSS in mice. The DAI score served as a valuable metric for assessing the severity of colitis, providing insightful guidance in clinical evaluations [12,24]. This score reflected the combined impact of the mouse’s weight change, fecal softness, and the degree of fecal bleeding, with a higher score indicating a more severe case of UC. According to Figure 2C, the DSS group had observably higher DAI score on the last day compared with the Normal group (*p* < 0.01). However, there was a markedly diminished in the DAI scores within the therapy group, and with statistically significant (*p* < 0.01). From what had been discussed above, ZOP-1 alleviated the mucosal injury caused by DSS and relieved its symptoms of diarrhea and bloody stool.

### 3.4. Body Weight and Organ Index

As shown in Figure 2D,E, the body weight change in mice in the Normal group, ZOP-1-L, and ZOP-1-H group was always on the rise, while the weight change in mice in the DSS group gradually flattened out and began to show a significant downward trend on the 13th day. On the last day of the experiment, the change in body weight in the DSS group was noticeably less than that in the Normal group (*p* < 0.01). ZOP-1-L, ZOP-1-H, and SAPS groups significantly alleviated this trend (*p* < 0.01). However, the ZOP-1-L and ZOP-1-H groups demonstrated a more significant improvement in mitigating weight loss among mice with UC, similar to the SASP group (*p* < 0.05). In a dose-dependent way, ZOP-1 reduced the weight loss caused by UC in mice.

Splenomegaly and thymus atrophy are common in mice with UC due to increased inflammation and decreased immunity. There were notable enlargements and reductions in the thymus and spleen in the DSS group (Figure 2F). In addition, after ZOP-1-L and ZOP-1-H treatment, the spleen volume and spleen coefficient of UC mice were decreased (*p* < 0.05, *p* < 0.01), and the thymus volume and thymus index were increased (*p* < 0.05, *p* < 0.01) (Figure 2G,H). This study revealed that ZOP-1 effectively rejuvenated the immune system in UC mice given DSS by suppressing thymus atrophy and splenomegaly, exhibiting an effect comparable to SAPS, with no notable statistical difference between the two treatments (*p* > 0.01).

Therefore, ZOP-1 alleviated the weight loss of UC mice and improved the immune system by relieving the swelling of the spleen and the atrophy of the thymus.

### 3.5. Morphological and Pathological Observation of Colon in Mice

**Figure 3:**
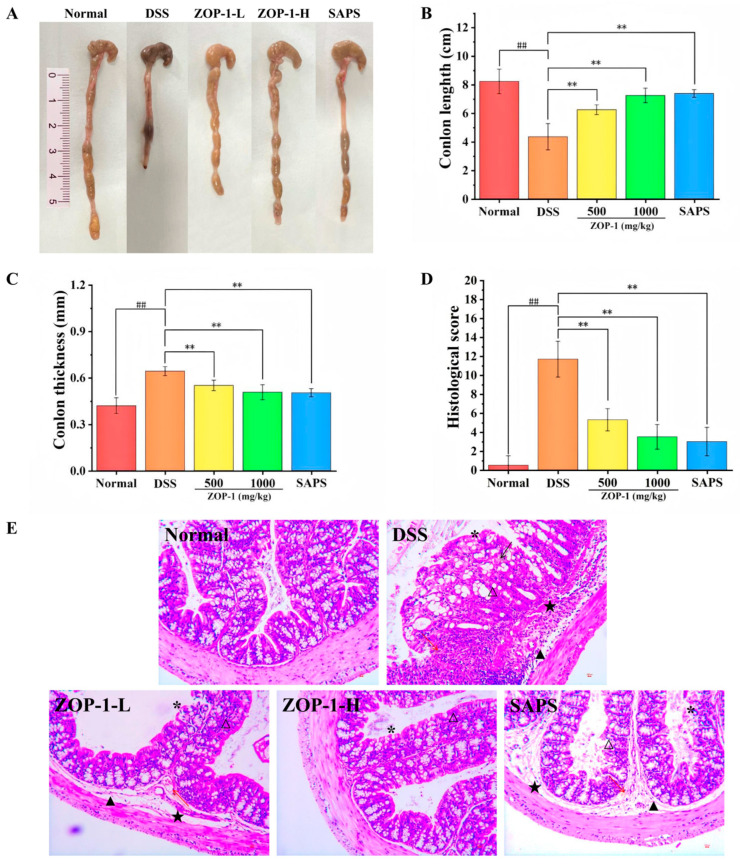
(**A**) Photographs of the colon; (**B**) colon length; (**C**) colon thickness; (**D**) histological score; In comparison to the Normal group; (**E**) HE staining of colons, the image magnification is ×200, and the black arrow, red arrow, △, *, ★, and ▲ indicate fibrosis in the lamina propria of the surface of the colon, inflammatory infiltration, crypt deformation, and goblet cell loss, thinning of the surface epithelium, thickening and vacuolization of the inner membrane, submucosal edema, respectively; ## *p* < 0.01; in comparison to the DSS group, ** *p* < 0.01 (n = 6).

The colonic length was also an indirect indicator of UC, which resulted in the thickening of the intestinal wall and shortening of the colon in mice [25,26]. As shown in Figure 3A,B, compared with the Normal group (8.23 ± 0.86 cm), the colon length of the DSS group was significantly reduced (6.25 ± 0.34 cm), which was statistically significant (*p* < 0.01). In contrast to the DSS group, the colon length of the ZOP-1-L group, ZOP-1-H group, and SAPS group was significantly increased, with average lengths of 8.45 ± 0.27, 9.51 ± 0.34 and 9.77 ± 0.53 cm, respectively. Meanwhile, ZOP-1-L and ZOP-1-H groups reversed the thickening of the colon wall in mice caused by UC (Figure 3C). These data indicated that ZOP-1 significantly inhibited the thickening of the intestinal wall and colonic contracture caused by DSS.

According to Figure 3E, the colon tissue of the Normal group contained glands that were grouped in an ordered fashion without the infiltration of inflammatory cells, and the mucosal epithelial structure was complete and visible. There was severe mucosal damage, crypt deformation, goblet cell loss, and inflammatory cell infiltration in the colon tissue of the DSS group. In the ZOP-1-L group, a slight degree of ulcer surface and inflammatory cell infiltration were observed, and mucosal lesions were alleviated to some extent. In the ZOP-1-H group, the colon tissue structure was intact, and the ulcer surface was not obvious. SASP group ulcer surface and Infiltration of inflammatory cells were not obvious. This suggested that ZOP-1 alleviated mucosal lesions caused by DSS and protected colon tissue. Histological scores based on HE (Figure 3D) staining showed that ZOP-1 played a dose–response role in alleviating colon injury caused by DSS. When ZOP-1 concentration reached 1000 mg/kg, the remission effect on colonic lesions was comparable to that of SAPS. In conclusion, ZOP-1 alleviated mucosal lesions caused by DSS and protected colon tissue.

### 3.6. Effects of ZOP-1 on Anti-Inflammatory Factors and Antioxidant Factors in Serum and Tissues of Mice with UC

**Figure 4:**
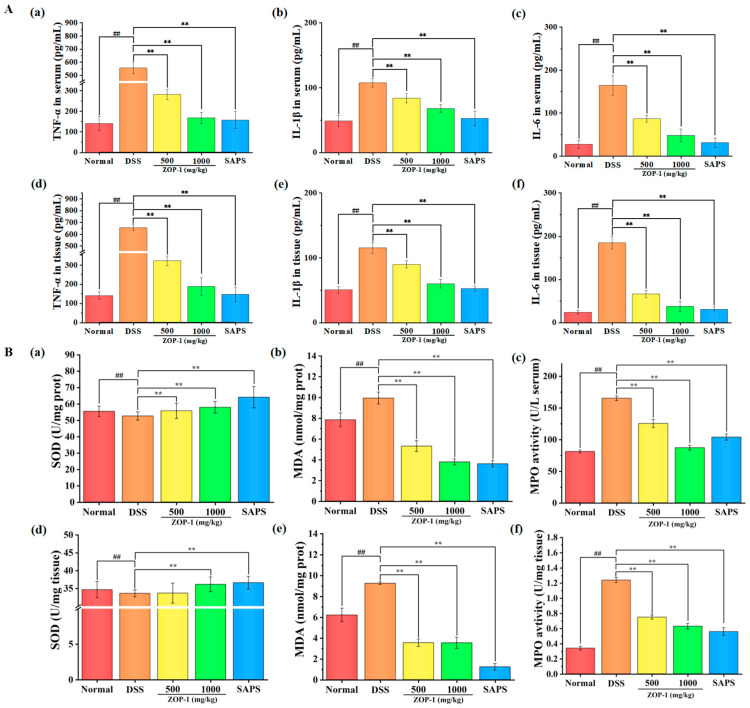
Protective effect of ZOP-1 on colonic injury in UC mice by an endoscope (n = 6 mice/group). (**A**): Effect of ZOP-1 on proinflammatory factors in DSS colitis mice. Serum: (**a**) TNF-α; (**b**) IL-1β; (**c**) IL-6. Tissue: (**d**) TNF-α; (**e**) IL-1β; (**f**) IL-6. (**B**): Effect of ZOP-1 on oxidative factors in DSS colitis mice. Serum: (**a**) SOD; (**b**) MDA; (**c**) MPO. Tissue: (**d**) SOD; (**e**) MDA; (**f**) MPO. In comparison to the normal group, ## *p* < 0.01; in comparison to the DSS group, ** *p* < 0.01 (n = 6).

It has been shown that colitis is related to the up-regulation and down-regulation of proinflammatory cytokines and anti-inflammatory cytokines [1]. As depicted in Figure 4A, the concentrations of inflammatory markers TNF-α, IL-1β, and IL-6 were notably elevated in both serum and colon tissue of the DSS group compared to the Normal group (*p* < 0.01). Conversely, the levels of these inflammatory factors were markedly reduced in the serum and colon tissues of the ZOP-1-L, ZOP-1-H, and SASP groups (*p* < 0.01). At the same time, oxidative stress was one of the key mechanisms of UC. The key hallmarks of oxidative stress involve the excessive generation of MDA and MPO, along with a decline in the levels of SOD [27]. According to Figure 4B, the DSS group’s serum and tissue SOD levels considerably dropped relative to the Normal group (*p* < 0.01), whereas MDA and MPO levels significantly increased (*p* < 0.01). However, ZOP-1 significantly reduced the oxidative stress reaction caused by DSS (*p* < 0.01). Therefore, ZOP-1 reduced the content of proinflammatory factors and oxidative stress to a certain extent, suggesting that ZOP-1 had obvious anti-inflammatory and antioxidant effects on DSS-induced UC. Purple sweet potato polysaccharides had similar relieving effects on colon inflammation [26].

### 3.7. Effects of ZOP-1 on Gut Microbiota

Metagenomics studies learn the genetic, functional, and ecological characteristics of microbial communities by directly analyzing the DNA of microorganisms in the environment [28]. In this study, most of the original data (>99%) had come from clean data, and the Q20(%) of the intestinal microorganisms in each component was more than 97% (Table S2). The results proved the reliability of metagenome sequencing data and could be used for subsequent research.

**Figure 5:**
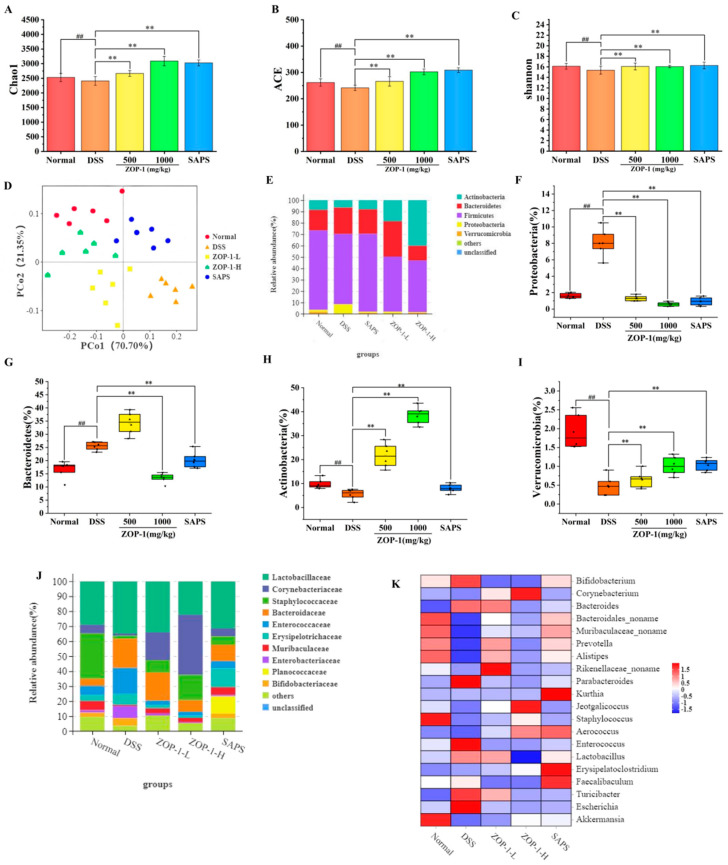
(**A**–**C**) alpha diversity indices of Chao1, ACE, and Shannon were observed; (**D**) PCoA analysis of OTU level; (**E**) histograms of hilus abundance between groups; (**F**–**I**) relative abundance of individual phyla; (**J**) histograms of genus abundance between groups; (**K**) genus-level species abundance heat map. (n = 6 mice/group). In comparison to the normal group, ## *p* < 0.01; in comparison to the DSS group, ** *p* < 0.01 (n = 6).

Chao1 and Shannon index reflect alpha diversity, and the greater the value, the higher the richness and diversity of intestinal microorganisms. In comparison to the Normal group, the Chao1 index (Figure 5A), ACE index (Figure 5B), and the Shannon index (Figure 5C) showed a significant decrease in the DSS group (*p* < 0.01), indicating a notable reduction in diversity. The mouse gut microbiota diversity index was positively correlated with the concentration of the ZOP-1 group. The gut microbiota species diversity and abundance in mice were reduced by DSS, in contrast, ZOP-1-L increased species richness and brought the intestinal flora diversity index in mice closer to that of the Normal group. In addition, the Coverage index of the 5 groups was high, with an average value of 0.999 and sequence similarity of N97%. The sequencing results better reflect the actual situation of intestinal flora in this study.

At the same time, principal coordinate analysis (PCoA) showed that DSS-induced colitis changed the composition and structure of intestinal microbiota [29]. PCoA analysis of OUT abundance across sample groups revealed marked differences, suggesting that the DSS group, as well as the ZOP-1-L, ZOP-1-H, and SAPS groups, had a distinct influence on the makeup and quantity of the gut microbiota. As can be seen from Figure 5D, the DSS group displayed a greater degree of dispersion compared to the Normal group, indicating that the microbial community structure changed more obviously. On the contrary, ZOP-1-L, ZOP-1-H, and SAPS groups were close to the Normal group, among which ZOP-1-H was the most similar. In conclusion, ZOP-1 showed obvious potential for the recovery of intestinal flora and structure in UC mice.

As shown in Figure 5E–I, the composition of intestinal flora was similar, mainly composed of *Actinobacteria*, *Bacteroidetes*, *Firmicutes*, *Proteobacteria*, *Verrucomicrobia*, and so on with different relative abundances. It was noteworthy that *Actinobacteria*, *Bacteroidetes*, and *Firmicutes* collectively comprised the majority (90%) of microbial composition. *Actinobacteria*, *Firmicutes*, and *Verrucomicrobia* were significantly reduced in number after the implementation of DSS (*p* < 0.05), but increased *Bacteroidetes* and *Proteobacteria* (*p* < 0.05). *Proteobacteria* was one of the primary pathogenic bacteria among them, which could induce excessive proinflammatory cytokines, thus accelerating the inflammatory process [30]. However, ZOP-1 effectively reversed the changes in the relative abundance of intestinal microflora caused by DSS. The calculated ratio of *Firmicutes*/*Bacteroides* (F/B) in the ZOP-1-H group (3.51) was higher than that in the DSS group (2.66). Thus, by regulating gut flora, ZOP-1 improved the inflammatory response of the body and played a certain preventive and therapeutic effect on DSS-induced UC. This result was consistent with the previous research results [31].

At the level of genus (Figure 5J,K), the abundance of *Bifidobacteriaceae*, *Bacteroidaceae*, *Enterococcaceae*, and *Erysipelotrichaceae* in mice modeled by DSS increased significantly (*p* < 0.05), while *Corynebacterium*, *Muribaculaceae* and *Staphylococcaceae* decreased significantly (*p* < 0.05). The increase in *Enterococcaceae* and the decrease in *Muribaculaceae* might lead to the overgrowth of pathogenic bacteria and the aggravation of intestinal inflammation [32]. The above results showed that DSS caused the imbalance of intestinal flora structure in mice. The intestinal microbiota of mice was changed after ZOP-1 treatment. *Muribaculaceae* abundance in the intestinal microbiota of mice with DSS-induced UC was considerably increased by ZOP-1 (*p* < 0.05).

The results were similar to those of [14]. In conclusion, we believe that ZOP-1 could alleviate the disruption of gut flora caused by UC.

### 3.8. Effects of ZOP-1 on Production of SCFAs

Multiple research endeavors have consistently demonstrated that SCFAs, notably butyric acid, and acetic acid, possess potent abilities in mitigating the manifestations of UC [33]. As shown in Table S3, compared with the Normal group (24.19 ± 0.04 mm), the total SCFA content in feces of UC mice induced by DSS decreased (22.26 ± 0.04 mm), especially the levels of acetic acid and butyric acid decreased (*p* < 0.01). Notably, ZOP-1 therapy significantly promoted the synthesis of these acids (*p* < 0.01), even exceeding the level observed in the normal group. This suggested that ZOP-1 effectively stimulated the generation of SCFAs. Hence, it might be that ZOP-1 played a role in UC by increasing the concentration of SCFAs in the intestinal tract.

### 3.9. Effect of ZOP-1 on the Expression of Occludin and ZO-1 Protein in Intestine

**Figure 6:**
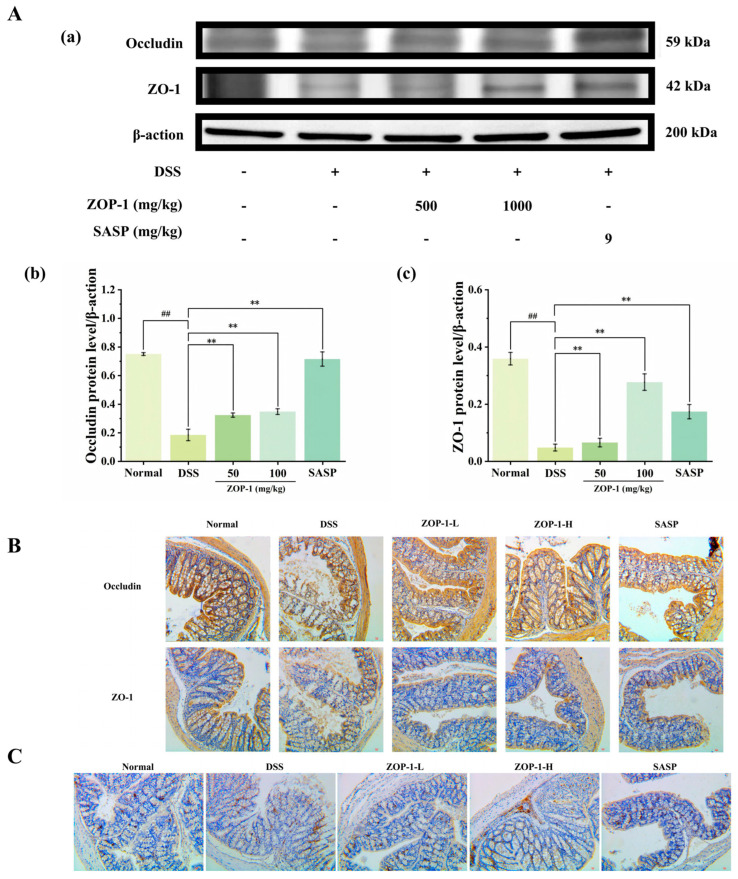
(**A**): The effect of ZOP-1 on TJs protein level: (**a**) Western blot; (**b**,**c**) density analysis. (**B**): Positive expression of Occludin and ZO-1 in colitis mice induced by DSS (Brown) (200·) (Scale, 100 µm). (**C**): Ly6G+ positive cells in colonic tissue stained with an immunohistochemical method (scale, 100 μm). ## *p* < 0.01; in comparison to the DSS group, ** *p* < 0.01 (n = 6).

The level of TJ protein was detected by Western blot and immunohistochemistry, and the effect of ZOP-1 on the intestinal barrier was discussed. An imbalance in the regulation of the epithelial junction complex and heightened susceptibility to colitis may have contributed to the decreased expression of ZO-1 and occludin proteins in the DSS group (*p* < 0.01), as demonstrated by Western blot analysis (Figure 6A), which indicated an impairment in the integrity of the colonic mucosal barrier. However, after the intervention of ZOP-1-L, ZOP-1-H, and SASP, the expression of Occludin protein and ZO-1 increased (*p* < 0.01). Meanwhile, the expression level of TJs protein was detected by immunohistochemistry, and the same result was obtained (Figure 6B). In conclusion, ZOP-1 increased the expression of TJ protein and improved intestinal permeability to some extent, suggesting that ZOP-1 played a therapeutic role by repairing the intestinal barrier.

The expression of Ly6G+ in the colon was evaluated by immunohistochemical staining. The results showed that the content of Ly6G+ in the colon of DSS-induced mice increased significantly, while the content of Ly6G+ in the colon of the ZOP-1-L group, ZOP-1-H group, and SASP group decreased significantly (*p* < 0.01). The ZOP-1-H group showed a higher inhibitory effect than the ZOP-1-L group, indicating that the inhibition was dose-dependent (Figure 6C). Except in the Normal group, varying degrees of crypt injury and goblet cell reduction occurred in the DSS group, ZOP-1-L group, ZOP-1-H group, and SASP group. However, after polysaccharide administration, these conditions were significantly improved. In conclusion, ZOP-1 inhibited the damage to intestinal mucus in mice with UC and thus played a preventive and therapeutic role.

### 3.10. Effect of ZOP-1 on Protein Expression in TLR4/NF-κB/MAPK Signaling Pathway in Mice with UC

**Figure 7:**
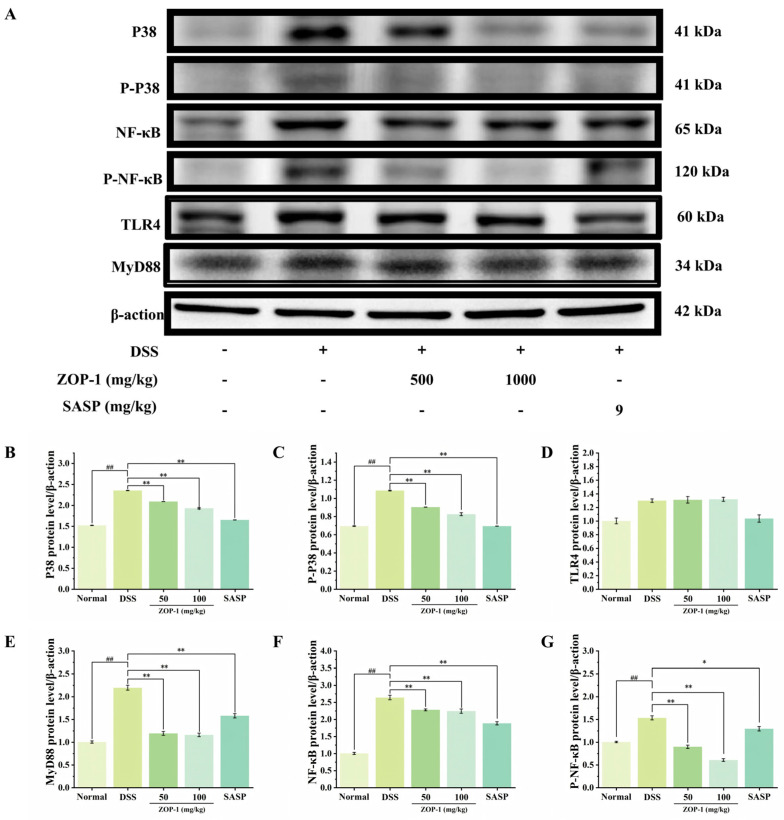
(**A**) The results of Western blot were detected in each group; (**B**–**G**) Effect of ZOP-1 on the gray level of protein expression in colonic tissue of mice with UC. In comparison to the Normal group, ## *p* < 0.01; in comparison to the DSS group, * *p* < 0.05, ** *p* < 0.01 (n = 6).

The activation of TLR4 transmits signals to transcription factors such as NF-κB through a series of signal molecules (such as MyD88) and then activates MAPK and other signal pathways, thus triggering inflammatory reactions [31]. The colon of the DSS group had considerably higher levels of TLR4 and NF-κB protein than the colon of the Normal group (Figure 7), suggesting that DSS caused an inflammatory immune response in the intestine. At the same time, the levels of MAPK protein P38 phosphorylation in the colon of mice belonging to the DSS group increased (*p* < 0.01), indicating that DSS led to the activation of the NF-κB and P38 MAPK signaling pathways. However, after the prognosis of ZOP-1, MyD88, p-NF-κB p65, P38 MAPK, and p-P38 MAPK protein expression levels all somewhat dropped (*p* < 0.01). Therefore, in mice with UC brought on by DSS, ZOP-1 could suppress the MyD88/NF-κB/MAPK signaling pathway to reduce the inflammatory response of colon tissue.

In conclusion, ZOP-1 had certain preventive and curative properties against UC in mice induced by DSS, and its mechanism of action might involve modulating the inflammatory response, mitigating tissue damage, and preserving the integrity and functionality of the intestine.

## 4. Discussion

**Figure 8:**
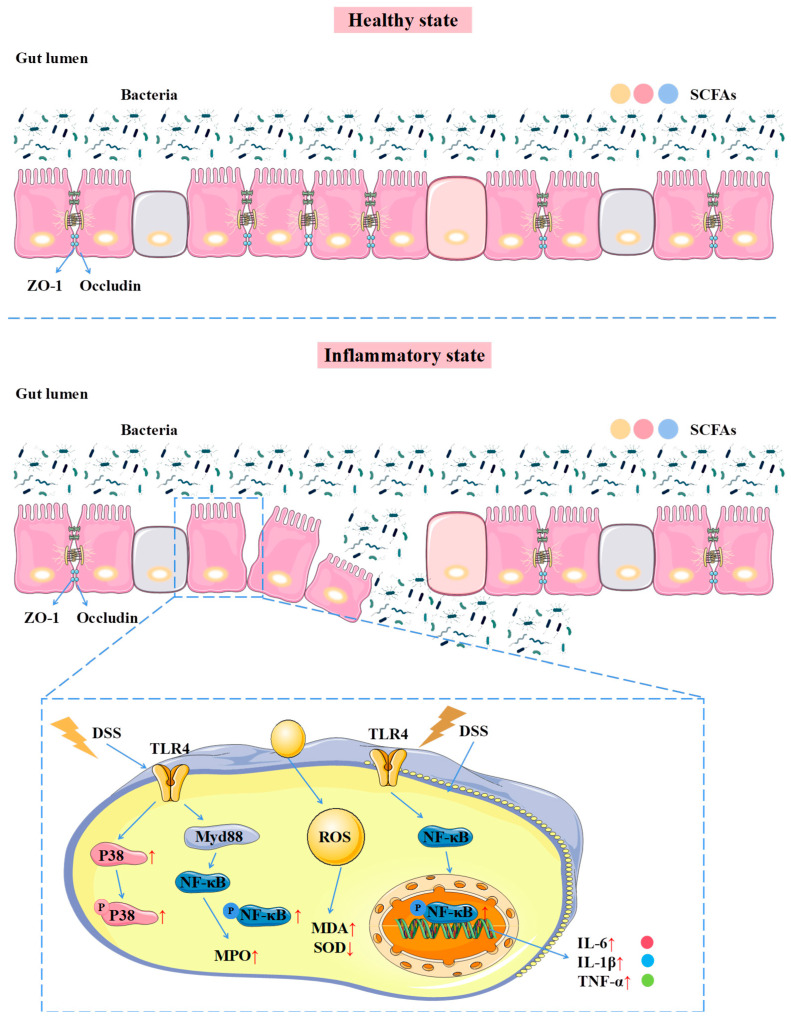
Prevention and treatment mechanism of ginger polysaccharide ZOP-1 on UC in mice [ZOP-1 can promote the production of intestinal SCFAs, raise the proportion of probiotics in the gut, improve the damage of intestinal epithelial cells, modulate inflammatory cytokines (marked decrease in TNF-α, IL-6, IL-1β levels), antioxidant factors (down-regulating MDA, MPO, and so on) in mice. Up-regulate SOD level and regulate TLR4/NF-κB/MAPK pathway to improve colonic tissue inflammation in mice with UC induced by DSS].

The incidence of UC is increasing year by year. The side effects of current clinical drugs and the heavy economic burden have made patients overwhelmed [34]. It is urgent to develop new safe and effective drugs to prevent and treat UC. Medicinal plants are one of the main sources of functional and active polysaccharides. Studies have shown that medicinal plant polysaccharides can effectively treat UC through anti-inflammatory, antioxidant, down-regulation of intestinal abnormal immune enhancement, regulation of intestinal flora, and other ways, with reliable efficacy [29,35]. Therefore, in this experiment, the DEAE-52 cellulose anion exchange column was used to separate and purify ZOP, and the washed refined polysaccharide ZOP-1 was obtained, and it was applied to study the mechanism of prevention and treatment of UC in vivo, and the mechanism of prevention and treatment of UC was speculated as shown in Figure 8.

In this study, DSS was used to construct a mouse model of ulcerative colitis. The UC mice induced by DSS will have symptoms such as weight loss, shortened colon, diarrhea, and bloody stool [34]. ZOP-1 significantly improved the symptoms of UC. Although the exact pathogenesis of UC is still unclear, more and more studies have shown that inflammatory diseases and epithelial barrier dysfunction are important triggers for the development and progress of UC [36]. Increased levels of inflammatory cytokines, such as TNF-α, IL-1β, and IL-6, in colon tissue worsens colon damage and causes a large number of inflammatory cells to accumulate in inflammatory tissues [1,23], and when tissues and organs are injured by inflammation, excessive oxygen free radicals will be generated. The activation of the TLR4/NF-κB signaling pathway is very important in inflammatory reactions, and its abnormal activation destroys the homeostasis of intestinal mucosa, which leads to further deterioration of intestinal inflammation [37]. The results showed that ZOP-1 significantly inhibited the secretion of TNF-α, IL-1β and IL-6 cytokines in the colon tissue of UC mice induced by DSS, reduced the contents of MDA and MPO (*p* < 0.01) and increased the content of SOD (*p* < 0.01), suggesting that ZOP-1 was closely related to inflammation and oxidation, and ZOP-1 inhibited the activation of TLR4/NF-κB signal pathway and reduced the occurrence of inflammatory reaction. In addition to causing damage to the colonic mucosa and increasing intestinal permeability, intestinal inflammation significantly alters the intestinal environment [1,23]. The epithelial barrier is an important physical barrier in the intestine, which is composed of the apical junction complex (AJC) and TJs [38]. Intestinal integrity and permeability are closely related to the expression level of TJs, and the abnormal expression of TJs in the intestine of mice with UC will aggravate the development of colitis [39]. Therefore, the contents of locking protein and ZO-1 in the small intestinal epithelium were determined. The results showed that ZOP-1 could increase the expression of TJs in the colon tissue of mice with ulcerative colitis, including locking protein and ZO-1.

Natural plant polysaccharides cannot be digested by the body and are mainly degraded by intestinal microorganisms in the colon to produce metabolites such as SCFAs. In the development of UC, polysaccharides are involved in the regulation of intestinal flora and the preservation of intestinal microenvironment homeostasis. Among them, acrylic acid can effectively relieve UC [33]. Butyric acid has the ability to control intestinal epithelial cell growth and proliferation while preserving the integrity of the intestinal epithelial cell barrier. Acetic acid, propionic acid, and butyric acid have the ability to alleviate intestinal inflammation by interacting with leukocytes and endothelial cells. Furthermore, they expedite the healing of intestinal mucosa by enhancing the expression of proteins such as Occludin and ZO-1. In addition, metagenomic sequencing results showed that ZOP-1 could ameliorate intestinal microbial disturbance in mice with UC induced by DSS to a certain extent. Among them, *Proteobacteria* is the main pathogen causing UC, which can promote the excessive production of proinflammatory cytokines [30]. After ZOP-1 treatment, the number of *Proteobacteria* in the intestinal tract of mice with UC was significantly decreased. Studies have found that *Firmicutes*/*Bacteroidetes* (F/B) value may be related to inflammatory diseases, the lower the F/B value, the milder the inflammatory response of the body [31]. The experimental results showed that ZOP-1 improved the inflammatory response of the body by regulating intestinal flora and had a certain preventive and therapeutic effect on UC induced by DSS.

To sum up, this study characterized the structure of ZOP-1, determined the effect of ZOP-1 on oxidative inflammatory factors, and discussed the effect of ZOP-1 on intestinal flora diversity and intestinal integrity, which provided a new strategy for ZOP-1 to treat UC. However, there are some limitations in the experiment, and the structure-activity relationship between ZOP-1 and the prevention and treatment of ulcerative colitis is still unclear, and needs further study. At the same time, the role of intestinal flora in the treatment of UC needs to be verified by fecal bacteria transplantation in subsequent experiments. In addition, the current experimental results are obtained from animal experiments and need more clinical data to support them.

## 5. Conclusions

This study showed that ZOP-1 regulated the expression of cytokines, reduced intestinal inflammation, enhanced intestinal integrity, regulated intestinal flora and SCFAs, and relieved UC.

## Figures and Tables

**Table 1:** MS results of methylation analysis of ZOP-1.

Linkage Types	Methylated Alditol Acetate	Retention Time (min)	Molar Ratio (%)	Mass Fragments (*m*/*z*)
A Araf-1	2,3,5-Me_3_-Araf	6.505	11.01	43, 71, 87, 101, 117, 129, 145, 161
B →4,6)-Glcp(1→	2,3-Me_2_-Glc*p*	7.023	5.17	43, 71, 85, 87, 99, 101, 117, 127, 159, 161, 201
C →4)-Glcp(1→	2,3,6-Me_3_-Glcp	9.995	78.69	43, 87, 99, 101, 113, 117, 129, 131, 161, 173, 233
D →3,6)-Galp(1→	2,4-Me_2_-Gal*p*	9.316	5.13	43, 87, 117, 129, 159, 189, 233

**Table 2:** Assignment of ^13^C and ^1^H NMR chemical shifts in ZOP-1.

Residues		Chemical Shifts δ (ppm)						
		C1/H1	C2/H2	C3/H3	C4/H4	C5/H5	C6/H6	CH3
A	-α-Araf-(1→	108.99/ 5.15	82.15/ 4.10	79.45/ 3.88	82.77/ 4.03	62.20/ 3.67	60.66/ 3.60	3.71
B	→4,6)-β-Glcp(1→	97.73/ 4.85	72.11/ 3.45	73.22/ 3.56	77.28/ 3.52	72.91/ 3.63	69.73/ 3.30	-/ -
C	→4)-β-Glcp(1→	95.78/ 4.52	71.60/ 3.58	73.38/ 3.90	76.92/ 3.44	71.26/ 3.82	60.57/ 3.80	-/ -
D	→3,6)-α-Galp(1→	91.86/ 5.12	69.71/ 4.24	77.75/ 3.89	74.11/ 3.61	74.29/ 3.54	75.22/ 4.40	-/ -

## Data Availability

The original contributions presented in the study are included in the article, further inquiries can be directed to the corresponding author.

## Supplementary Materials

The following supporting information can be downloaded at: https://www.mdpi.com/article/10.3390/foods14050753/s1, Table S1: Mayo Endoscopic Scoring Criteria (n = 6 mice/group); Table S2: Metagenomic sequencing data of intestinal microbes from DSS UC mice; Table S3: Analysis of SCFAs in cecum contents of UC mice.

## References

[1] Kanwal S., Joseph T.P., Aliya S., Song S., Saleem M.Z., Nisar M.A., Wang Y., Meyiah A., Ma Y., Xin Y. (2020). Attenuation of DSS induced colitis by *Dictyophora indusiata* polysaccharide (DIP) via modulation of gut microbiota and inflammatory related signaling pathways. J. Funct. Foods.

[2] Su Y., Cui Z.Y., Chen C., Yang X.Y., Jiang Y.M., Zhang W., Zhang Y., Man C.X. (2025). Lactobacillus paracasei JY062 with its exopolysaccharide ameliorates intestinal inflammation on DSS-induced experimental colitis through TLR4/MyD88/NF-κB signaling pathway. Food Biosci..

[3] Du L., Ha C. (2020). Epidemiology and pathogenesis of ulcerative colitis. Clin. Gastroenterol..

[4] Feng X., Li H.Y., Yao H.L., Ou Y.H., Chan S.C., Wang S.P., Li H.J., Lan W.J. (2025). A galacturonic acid-rich polysaccharide from *Citrus medica* ‘fingered’ alleviated the dextran sulfate sodium-induced ulcerative colitis. Int. J. Biol. Macromol..

[5] Lavelle A., Sokol H. (2020). Gut microbiota–derived metabolites as key actors in inflammatory bowel disease. Nature reviews. Gastroenterol. Hepatol..

[6] Zheng L., Chu T.J., Sun X., Shen Z., Hou D.B., Zhang Z.H., Sun J.L., Liu Y.H., Li J., Bian Y.F. (2025). Polyphenols-rich *Portulaca oleracea* L. (purslane) alleviates ulcerative colitis through restiring the intestinal barrier, gut microbiota and metabolites. Food Chem..

[7] AliKhan M.W., Sherwani S., Alshammari M.H.E., Alsukaibi A.K.D., AliKhan W., Haque A., Alenezi K.M., Shahab U. (2024). Pharmacological Activities of Zingiber officinale Roscoe: Inhibition of HSA Protein Glycation, Structure Stability and Function Restoration. Pharmaceuticals.

[8] Liao D., Cheng C., Liu J., Zhao L.Y., Huang D.C., Chen G.T. (2020). Characterization and antitumor activities of polysaccharides obtained from ginger (*Zingiber officinale*) by different extraction methods. Int. J. Biol. Macromol..

[9] Hu Y.Q., Li Z., Yang J., Bai R.B., Marchioni E., Zhao M.J., Li Z. (2024). Anti-inflammatory mechanism of *Houttuynia cordata* polysaccharides against ulcerative colitis based on multi-omics conjoint analysis. Int. J. Biol. Macromol..

[10] Wang Z., Li Z., Wang H., Wu Q., Geng Y. (2024). Effects of *Pine Pollen* Polysaccharides and Sulfated Polysaccharides on Ulcerative Colitis in Mice by Regulating Th17/Treg. Foods.

[11] Li R.X., Wang H., Wang Q.J., Zhang Z.Q., Wang L. (2024). Acid-assisted polysaccharides extracted from *Asparagus cochinchinensis* protect against Alzheimer’s disease by regulating the microbiota-gut-brain axis. Front. Nutr..

[12] Salem B.M., Lakkany E.M.N., Hammam A.O., Din S.H.S. (2025). Bacillus clausii spores maintain gut homeostasis in murine ulcerative colitis via modulating microbiota, apoptosis, and the TXNIP/NLRP3 inflammasome cascade. Toxicol. Rep..

[13] Ning E.J., Sun C.W., Wang X.F., Chen L., Li F.F., Zhang L.X., Wang L.P., Ma Y.N., Zhu J., Li X. (2024). *Artemisia argyi* polysaccharide alleviates intestinal inflammation and intestinal flora dysbiosis in lipopolysaccharide-treated mice. Food Med. Homol..

[14] Yang W., Ren D., Zhao Y., Liu L., Yang X. (2021). Fuzhuan brick tea polysaccharide improved ulcerative colitis in association with gut microbiota-derived tryptophan metabolism. J. Agric. Food Chem..

[15] Niu Z.Q., Liu Y.N., Zhou D.Y., Feng J.L., Hu Y.Y., He Z.L., Shen T., Piao J.P., Wu H.F., Hu W.C. (2025). Ginsenoside F2 from the leaves of Panax ginseng alleviates DSS-induced ulcerative colitis: An in silico analysis and in vivo investigation. Ind. Crops Prod..

[16] Lu H., Shen M., Chen T., Yu Y., Chen Y., Yu Q., Chen X., Xie J. (2022). Mesona chinensis benth polysaccharides alleviate DSS–induced ulcerative colitis via inhibiting of TLR4/MAPK/NF–κB signaling pathways and modulating intestinal microbiota. Mol. Nutr. Food Res..

[17] Jing Y.S., Cheng W.J., Li M.S., Zhang Y.M., Pang X.Y., Qiu X.Y., Zheng Y.G., Zhang D.S., Wu L.F. (2023). Structural Characterization, Rheological Properties, Antioxidant and Anti-Inflammatory Activities of Polysaccharides from *Zingiber officinale*. Plant Foods Hum. Nutr..

[18] Xu L.S., Zhang Y., Wang L. (2016). Structure characteristics of a water-soluble polysaccharide purified from dragon fruit (*Hylocereus undatus*) pulp. Carbohydr. Polym..

[19] Song Y., Zhu M., Hao H.L., Deng J., Li M.Y., Sun Y.M., Yang R.L., Wang H., Huang R.M. (2019). Structure characterization of a novel polysaccharide from Chinese wild fruits (*Passiflora foetida*) and its immune-enhancing activity. Int. J. Biol. Macromol..

[20] Zhu H.Q., Ding X., Hou Y.L., Li Y.M., Wang M. (2018). Structure elucidation and bioactivities of a new polysaccharide from Xiaojin Boletus speciosus Frost. Int. J. Biol. Macromol..

[21] Jing Y.S., Li M.S., Li Y.Q., Ma T., Qu Y., Hu B.B., Xie Y.H., Li Z.W. (2024). Structural characterization and anti-fatigue mechanism based on the gut-muscle axis of a polysaccharide from *Zingiber officinale*. Int. J. Biol. Macromol..

[22] Laroui H., Ingersoll S.A., Liu H., Baker M.T., Ayyadurai S., Charania M.A., Laroui F., Yan Y., Sitaraman S.V., Merlin D. (2012). Dextran sodium sulfate (DSS) induces colitis in mice by forming nano–lipocomplexes with medium–chainlength fatty acids in the colon. PLoS ONE.

[23] Okabe M., Yamamoto S.J., Kitamoto H., Kuwada T., Shiokawa M., Seno H.S. (2024). Sa1808 ANTI-INTEGRIN AVB6 ANTIBODY IS A USEFUL PREDICTIVE BIOMARKER FOR RELAPSE OF ULCERATIVE COLITIS IN PATIENTS WITH MAYO ENDOSCOPIC SUBSCORE 0. Gastroenterology.

[24] Xu J., Tang C.K., Ud D.A., Lu Y., Ma X.Y., Zhang T., Wu J.Q., Du Z.Q., Luo P., Wu J.B. (2023). Oligosaccharides of Polygonatum Cyrtonema Hua ameliorates dextran sulfate sodium-induced colitis and regulates the gut microbiota.Biomed. Pharmacother.

[25] Zhang S.L., Zhang S.Z., Wu Z.Y., Guo B.T., Li J., Huang X.Q., Zhang F.M., Li M.Y., Yang P.C., Zheng X.B. (2024). Activation of free fatty acid receptors, FFAR1 and FFAR4, ameliorates ulcerative colitis by promote fatty acid metabolism and mediate macrophage polarization. Int. Immunopharmacol..

[26] Sun J., Chen H., Kan J., Gou Y., Liu J., Zhang X., Wu X., Tang S., Sun R., Qian C. (2020). Anti–inflammatory properties and gut microbiota modulation of an alkali–soluble polysaccharide from purple sweet potato in DSS–induced colitis mice. Int. J. Biol. Macromol..

[27] Liu C., Hua H.Y., Zhu H.K., Cheng Y.L., Guo Y.H., Yao W.R., Qian H. (2021). Aloe polysaccharides ameliorate acute colitis in mice via Nrf2/HO-1 signaling pathway and short-chain fatty acids metabolism. Int. J. Biol. Macromol..

[28] Guo L., Zhang D., Fu S., Zhang J., Zhang X., He J., Peng C., Zhang Y., Qiu Y., Ye C. (2021). Metagenomic sequencing analysis of the effects of colistin sulfate on the pig gut microbiome. Front. Vet. Sci..

[29] Yang X., Wei S., Lu X., Qiao X., Simal-Gandara J., Capanoglu E., Woźniak Ł., Zou L., Cao H., Xiao J. (2021). A neutral polysaccharide with a triple helix structure from ginger: Characterization and immunomodulatory activity. Food Chem..

[30] Tsai Y.S., Wei C.T., Liang C.M., Wu C.K., Tsai M.C., Hu W.H., Huang P.Y., Chen C.H., Yuan H.K., Yao C.C. (2024). Alternations of the gut microbiota and the Firmicutes/Bacteroidetes ratio after biologic treatment in inflammatory bowel disease. J. Microbiol. Immunol. Infect..

[31] Guo C., Guo D., Fang L., Sang T., Wu J., Guo C., Wang Y., Wang Y., Chen C., Chen J. (2021). *Ganoderma lucidum* polysaccharide modulates gut microbiota and immune cell function to inhibit inflammation and tumorigenesis in colon. Carbohydr. Polym..

[32] Zhao X.Q., Wang L., Zhu C.L., Xue X.H., Xia X.J., Wu X.L., Wu Y.D., Liu S.Q., Zhang G.P., Bai Y.Y. (2022). Oral Administration of the Antimicrobial Peptide Mastoparan X Alleviates Enterohemorrhagic Escherichia coli-Induced Intestinal Inflammation and Regulates the Gut Microbiota. Probiotics Antimicrob. Proteins.

[33] Hu J., Mei Y., Zhang H., Li J., Zhang M., Li Y., Yang W., Liu Y., Liang Y. (2024). Ameliorative effect of an acidic polysaccharide from *Phellinus linteus* on ulcerative colitis in a DSS-induced mouse model. Int. J. Biol. Macromol..

[34] Ma J., Yue S., Liu Y., Gong L., He P., Yang Y., Fu Z., Han D., Hu Q., Liao F. (2024). Fucoxanthin ameliorates ulcerative colitis by maintaining the epithelial barrier via blocking JAK2/STAT3 signaling pathway. Toxicol. Appl. Pharmacol..

[35] Li J.Z., Li Q.K., Wu Q.H., Gao N., Wang Z.H., Yang Y., Shan A.S. (2023). Exopolysaccharides of Lactobacillus rhamnosus GG ameliorate Salmonella typhimurium-induced intestinal inflammation via the TLR4/NF-κB/MAPK pathway. J. Anim. Sci. Biotechnol..

[36] Jiang L., Ma X., Yan Q., Pu D., Fu X., Zhang D. (2024). Dihydromyricetin/montmorillonite intercalation compounds ameliorates DSS-induced colitis: Role of intestinal epithelial barrier, NLRP3 inflammasome pathway and gut microbiota. Int. J. Pharm..

[37] Yin W., Liu M., Jin Z., Hao Z., Liu C., Liu J., Liu H., Zheng M., Cai D. (2025). Ameliorative effects of insoluble dietary fiber and its bound polyphenols from adzuki bean seed coat on acute murine colitis induced by DSS: The inflammatory response, intestinal barrier and gut microbiota. Int. J. Biol. Macromol..

[38] Ivanov A.I., Lechuga S., Marino-Melendez A., Naydenov N.G. (2022). Unique and redundant functions of cytoplasmic actins and nonmuscle myosin II isoforms at epithelial junctions. Ann. N. Y. Acad. Sci..

[39] Zheng B., Ying M., Xie J., Chen Y., Wang Y., Ding X., Hong J., Liao W., Yu Q. (2020). A *Ganoderma atrum* polysaccharide alleviated DSS–induced ulcerative colitis by protecting the apoptosis/autophagy–regulated physical barrier and the DC–related immune barrier. Food Funct..

