# Supplementary material for: Structure Characterization and Treatment Effect of Zingiber officinale Polysaccharide on Dextran Sulfate Sodium-Induced Ulcerative Colitis
Source: Foods. 2025 Feb 23;14(5):753. doi: 10.3390/foods14050753 (PMC11899033; doi:10.3390/foods14050753)
Supplement: Supplementary file 1 [file foods-14-00753-s001.zip › foods-3443695-supplementary.pdf]

Table S1. Mayo Endoscopic Scoring Criteria (n=6 mice/group).

Scoring items	Score value (points)
Mucosal disease is normal or inactive	0
Mild injury (erythema, reduced blood vessels, mild fragility)	1
Moderate damage (marked erythema, loss of vascular pattern, brittle, erosion)	2
Severe injury (spontaneous bleeding, extensive ulcers)	3

Table S2 Metagenomic sequencing data of intestinal microbes from DSS UC mice.

Sample	Rawreads(bp)	Clean data (bp)	Q20 (%)	n (%)	GC (%)
Normal	11104794900	11081762875	10813203553 (97.37%)	85401 (0.0%)	4835496654 (43.54%)
DSS	9958306200	9939905716	9743109038 (97.84%)	54114 (0.0%)	4312396733 (43.3%)
ZOP-1-L	11719958700	11677175168	11402044003 (97.29%)	93470 (0.0%)	5281880237 (45.06%)
ZOP-1-H	11875473600	11853055076	11553313002 (97.29%)	90933 (0.0%)	5374780804 (45.26%)
SASP	12052706700	12029890198	11736503566 (97.38%)	92605 (0.0%)	5280149868 (43.8%)

Note: Q20 (%) represents the proportion of identical Q20 data inside the clean data; n (%) is the percentage of N bases in the clean data. G and C base percentages in clean data are represented by GC (%).

Table S3 Analysis of SCFAs in cecum contents of UC mice.

Group	Dose (mg/kg)	Acetic acid (mM)	Propionic acid (mM)	Butyric acid (mM)	Isovaleric acid (mM)	Valeric acid (mM)	Total content (Mm)
Normal	--	9.19±0.16	10.85±0.08	1.63±0.04	1.06±0.18	1.46±0.04	24.19±0.04
DSS	--	8.36±0.05##	10.35±0.12	1.23±0.19##	0.98±0.01##	1.34±0.06##	22.26±0.04##
ZOP-1-L	500	13.66±0.13**	12.15±0.01**	2.49±0.01**	1.24±0.18**	2.06±0.01**	31.60±0.02**
ZOP-1-H	1000	13.82±0.13**	15.14±0.01**	2.65±0.01**	1.37±0.18**	2.16±0.01**	35.14±0.06**
SASP	50	13.18±0.04**	11.28±0.01**	1.89±0.18**	1.05±0.04**	1.47±0.01**	28.87±0.01**

Note: In comparison to the DSS group, $^*p < 0.05$ $^*p < 0.01$; In comparison to the Normal group, $^\#p < 0.05$ $^{\#\#}p < 0.01$.
